# Idiopathic true aneurysm of the proximal radial artery with the appearance of an enlarging mass: A case report

**DOI:** 10.1016/j.ijscr.2023.108343

**Published:** 2023-05-29

**Authors:** Pezhman Kharazm, Nemat Nematollahi, Farhad Kor, Iman Shakeri, Shahryar Alizadeh, Mahshid Mehrjerdian

**Affiliations:** a Clinical Research Development Center, 5 Azar Hospital, Golestan University of Medical Sciences, Gorgan, Iran; b Dr Alizadeh Laboratory, Gorgan, Iran; c Neonatal and Children's Health Research Center, Golestan University of Medical Sciences, Gorgan, Iran

**Keywords:** True aneurysm, Radial artery, Mass, Case report

## Abstract

**Introduction and importance:**

Aneurysm of the radial artery is one of the rare cases in the field of vascular surgery, and its diagnosis and management is one of the challenges in this era. Without proper diagnosis and treatment, it may affect the patient's lifestyle and reduce the quality of life because of pain and limitation in activity. Importantly, it may cause limb or life threatening complications including arterial thromboembolism and aneurysm rupture.

**Case presentation:**

A 50-year-old woman was presented with a true proximal right radial artery aneurysm with the manifestation of an enlarging mass since 2 years ago, which had gradually become painful. The radiologic investigations revealed the presence of an aneurysm in the proximal radial artery. The patient underwent surgery consisting of ligation of the radial artery proximal and distal to the aneurysm and excision of the aneurysm sac. Pathologic examination confirmed the diagnosis.

**Clinical discussion:**

Any enlarging mass in the pathways of arterial branches can be arterial aneurysm, even if not pulsatile. Prompt physical examination and imaging modalities can help for diagnosis and decision making for either segmental arterial resection or vascular reconstruction.

**Conclusion:**

Radial artery aneurysm, as a rare but potentially devastating arterial disease should be in differential diagnosis of any forearm mass and managed promptly.

## Highlights

• Radial artery aneurysm is very rare in proximal part of the artery.
• Diagnosis of radial artery aneurysm requires a strong clinical suspicion.
• Radial artery aneurysms need to be treated because of compressive symptoms and probability of limb ischemia.
• When palmar arc is intact, the radial artery aneurysm can be excised and inflow/outflow branches ligated.

## 1 Introduction

In the upper limbs, after the subclavian, brachial, and ulnar arteries, the radial artery is the least affected artery by the aneurysmal disease, and the incidence of true aneurysm of the radial artery is very rare, and rare cases have been reported [1]. Almost all reported cases of radial artery aneurysms were located in the distal region of the artery and no case is reported in the proximal region of the artery.

The normal diameter of the radial artery is 2‐3 mm, and if there is a segmental dilatation of the artery more than 1.5 times of the normal diameter, it is defined as aneurysm [2,3]. Most radial artery aneurysms are pseudoaneurysms that develop secondary to interventions performed on this artery for angiographic purposes. These interventions include invasive endovascular procedures, invasive arterial pressure monitoring, or in injecting drug abusers [4,5].

On the other hand, true aneurysms are arterial dilatation that includes all components of the arterial wall, which usually occurs due to the weakening of the arterial wall and is mostly caused by repeated blunt trauma, arteriosclerotic conditions, metabolic and congenital diseases, or with connective-tissue disorders and vasculopathies [5,6].

In this case report, a true idiopathic aneurysm presenting as an enlarging mass in the proximal of the radial artery is presented. Also, diagnostic investigations and surgical treatment performed for the patient and one month follow up are reported.

The study has been reported in line with the SCARE criteria [7].

## 2 Case presentation

A 51-year-old female patient came to the surgery clinic with the complaint of forearm mass. She had noticed the mass 2 years ago on the volar aspect of her right forearm. It was small at first, but gradually enlarged. Since the last three months, the mass had increased significantly in size, and pain had also been added to the symptoms which limited the patient's daily activities.

The patient denied any trauma to the mass area and had no history of upper limb surgery. Apart from hypercholesterolemia, which was treated with atorvastatin tablets (20 mg/daily), she had no history of other underlying diseases such as diabetes, hypertension, cardiovascular disease, or connective tissue diseases. The patient was not taking any other medicine except atorvastatin tablets. She had no history of smoking or drug abuse, and she did not mention a positive family history of aneurysmal diseases. In the examinations performed, the patient's vital signs were normal. In the examination of the upper limb, the radial artery pulses of the upper limbs were palpable and strong. The Allen's test revealed patent palmar arch and normal ulnar artery.

The mass was located in the superior third of the volar aspect of the forearm and no erythema or cutaneous manifestations were seen on surrounding skin. The mass had a size of 3 × 3 cm and was tender to the palpation and had mild pulsation.

**Fig. 1:**
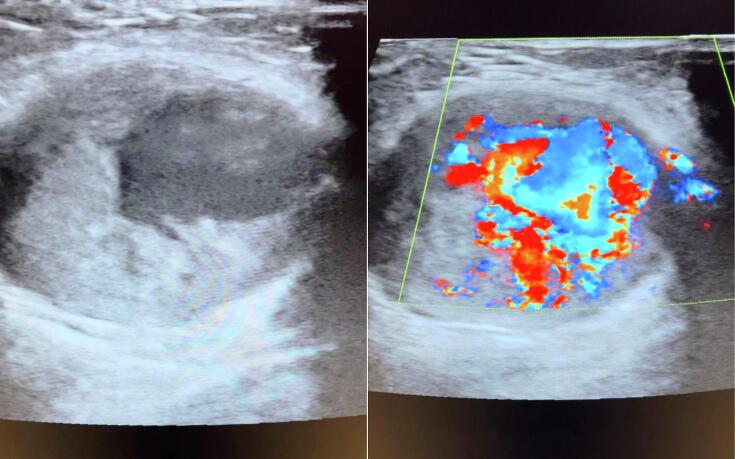
Gray scale and color Doppler sonography indicated a pulsating aneurysm with central regions of turbulent flow and peripheral solid thrombosis.

**Fig. 2:**
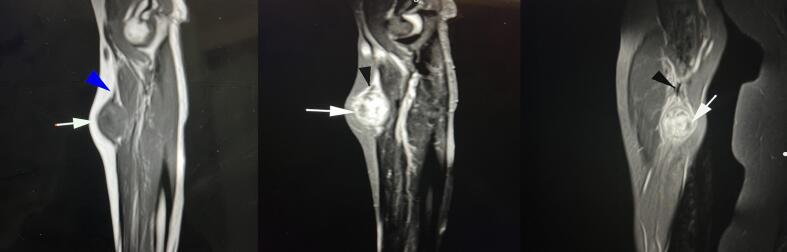
Sagittal T1 (a), sagittal PD fat saturation(b), and coronal PD fat saturation (c) MR images show well-defined soft tissue mass which is isosignal in T1 and high signal in T2. it shows Some T1 high signal components. Note also hypo signal components due to turbulent flow in all images (arrows). signal void radial artery (arrowheads)could also be visible, reaching this partially thrombosed aneurysm.

In laboratory work-up, blood, and inflammatory factors were normal and there were no signs of systemic diseases. In the diagnostic workup, a Doppler ultrasound of the upper limb and the mass was performed, which showed an aneurysm with a pulse with a turbulent central flow and peripheral solid thrombosis (Fig. 1). MRI was requested for the patient as shown in Fig. 2.

**Fig. 3:**
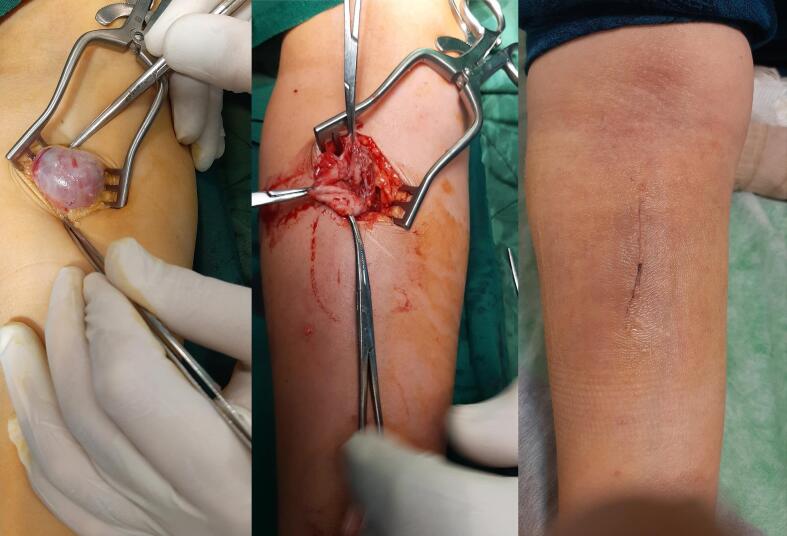
Radial artery aneurysm sac (a), dissection and repair of true radial artery aneurysm (b), the incision 3 weeks after operation (c).

**Fig. 4:**
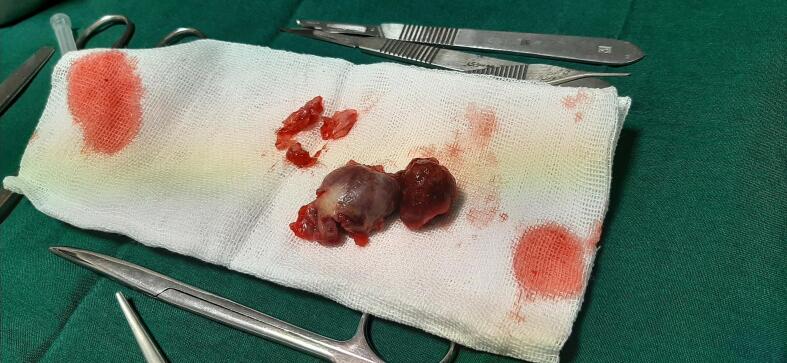
Resected true radial artery aneurysm and containing thrombosis.

**Fig. 5:**
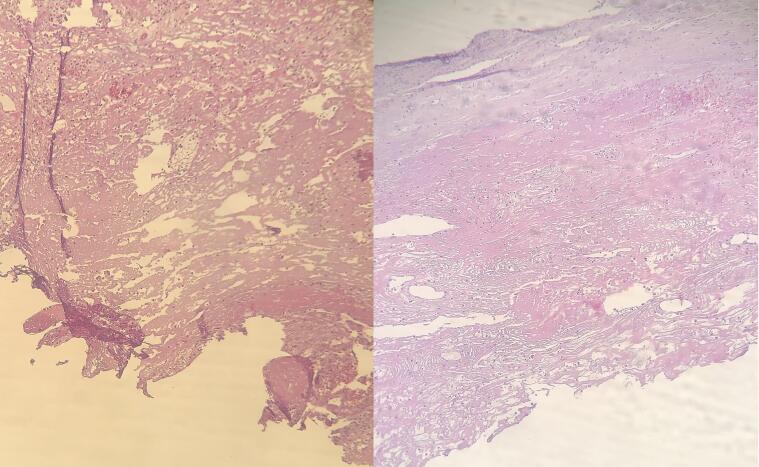
Severe degeneration of different layers of the arterial wall with infiltration of histiocytes is present.

Initially, according to the examinations and the results of the imaging studies, the diagnosis of radial artery pseudoaneurysm was made, and due to the possibility of complications such as arterial thrombosis or aneurysm rupture, the patient scheduled for surgery by a vascular surgeon. Under general anesthesia, proximal and distal controls of radial artery were obtained through a vertical incision just over the mass, extending proximally and distally. The aneurysm sac was dissected from the surrounding tissues carefully and observed. It was a true aneurysm (Fig. 3). Considering normal preoperative Allen's test and color Doppler result, the radial artery proximal and distal to the aneurysm was ligated, and the aneurysm sac was excised. After hemostasis and confirmation of normal hand perfusion, the incision was closed in anatomic layers (Fig. 3). The removed sac and aneurysm were sent for pathologic examination (Fig. 4). Pathologic examination revealed degeneration of arterial wall layers and histiocyte infiltration (Fig. 5). After the surgery, the patient underwent a 30-day follow-up and had good progress after the operation, and no ischemic or neurological defects were seen.

## 3 Discussion

Aneurysm of upper limb arteries is a very rare finding in the field of surgery, of which radial artery aneurysm is the rarest. The rarity of this condition is attributed to the fact that the radial artery has a small lumen, and because vessels with a small lumen require higher pressures to become aneurysmal, there is a small chance of aneurysm formation in radial artery [6,8]. Such aneurysms are associated with diagnostic and therapeutic challenges due to their low prevalence, and on the other hand, delaying surgical intervention in these cases is not recommended due to the risk of thromboembolism or aneurysm rupture [9].

As in this case, the most common manifestation of radial artery aneurysm is a pulsating mass with or without pain. If the adjacent nerve is compressed due to the enlargement of the aneurysm, the patient may also have symptoms of paresthesia. In case of rupture or thromboembolism, upper limb ischemia symptoms such as severe pain, pallor, and coldness of the limb may occur. Aneurysms that are larger and located in the proximal part of the upper limb have a higher risk of complications [10].

The diagnosis of a true radial artery aneurysm is made by clinical examination and radiologic studies and is confirmed by pathological examination after surgery [11].

Doppler ultrasound and CT angiography are methods commonly used to diagnose aneurysms. The treatment of radial artery aneurysms is still controversial. Current treatment guidelines are ambiguous between surgery and conservative treatment, and the choice of surgery depends on size, location, and symptoms [12].

There is no definition of clinical follow-up without surgical treatment. Therefore, surgical repair of the aneurysm is always indicated due to the risk of thrombotic and micro embolic complications that can lead to distal ischemia. Surgical options vary from proximal and distal ligation of the artery and then removing the aneurysm sac to revascularization methods or bypass with venous graft, and are selected according to the conditions of the aneurysm, the patient, the available surgical facilities, and the experience of the surgeon.

Finally, it should be noted that the patient's full consent was obtained for the publication of this article and images and no conflict of interest is present between authors.

## Ethical approval

This study was approved by the Golestan University of Medical Sciences Research Ethics Committee with the following ethics code: https://ethics.research.ac.ir/IR.GOUMS.REC.1401.547.

Date of approval: 07/Feb/2023

## Funding

There is no funding source for this study.

## CRediT authorship contribution statement

Dr. Pezhman Kharazm, vascular surgeon and the patient's corresponding physician.

Dr. Nemat Nematollahi, radiologic consultant.

Dr. Farhad Kor, literature reviewer.

Dr. Iman Shakeri, assistant surgeon and paper writer.

Dr. Shahryar alizade, Pathologic evaluation of the specimen and picture provider.

Dr. Mahshid Mehrjerdian, supervision of pathologic report.

## Guarantor

Dr. Pezhman Kharazm

## Consent

Written informed consent was obtained from the patient for publication of this case report and accompanying images. A copy of the written consent is available for review by the Editor-in-Chief of this journal on request.

## Conflict of interest statement

There is no conflict of interest between authors.
